# Application of region-based video surveillance in smart cities using deep learning

**DOI:** 10.1007/s11042-021-11468-w

**Published:** 2021-12-27

**Authors:** Asma Zahra, Mubeen Ghafoor, Kamran Munir, Ata Ullah, Zain Ul Abideen

**Affiliations:** 1 Department of Computer Science, COMSATS University Islamabad, Islamabad, Pakistan; 2 School of Computer Science, University of Lincoln, Lincoln, UK; 3 Department of Computer Science and Creative Technologies (CSCT), University of the West of England (UWE), Bristol, UK; 4 Department of Computer Science, National University of Modern Languages, Islamabad, Pakistan

**Keywords:** Deep learning, Video surveillance, Surveillance cameras, Smart cities and towns, Smart city applications

## Abstract

Smart video surveillance helps to build more robust smart city environment. The varied angle cameras act as smart sensors and collect visual data from smart city environment and transmit it for further visual analysis. The transmitted visual data is required to be in high quality for efficient analysis which is a challenging task while transmitting videos on low capacity bandwidth communication channels. In latest smart surveillance cameras, high quality of video transmission is maintained through various video encoding techniques such as high efficiency video coding. However, these video coding techniques still provide limited capabilities and the demand of high-quality based encoding for salient regions such as pedestrians, vehicles, cyclist/motorcyclist and road in video surveillance systems is still not met. This work is a contribution towards building an efficient salient region-based surveillance framework for smart cities. The proposed framework integrates a deep learning-based video surveillance technique that extracts salient regions from a video frame without information loss, and then encodes it in reduced size. We have applied this approach in diverse case studies environments of smart city to test the applicability of the framework. The successful result in terms of bitrate 56.92%, peak signal to noise ratio 5.35 bd and SR based segmentation accuracy of 92% and 96% for two different benchmark datasets is the outcome of proposed work. Consequently, the generation of less computational region-based video data makes it adaptable to improve surveillance solution in Smart Cities.

## Introduction

Video surveillance is a key element for groundwork of a balanced smart city. Video data from all-over the smart city is collected from cameras acting as visual sensors. The collected visual data can be used to analyze objects and their characteristics from recorded scenes. There are several applications which helps to structure basic services of smart city such as smart traffic management and enhanced public security [5, 14]. These applications require uninterrupted streaming of salient visual data even when the bandwidth of transmitting network is low in capacity. For instance, in case of large-scale video analysis for tracking propose the suspected salient region (S-R) of vehicle, person or road is required in high quality for efficient video surveillance [14]. In order to ensure video transmission over limited bandwidth, video compression is mandatory where quality of salient regions is also guaranteed. In literature, a number of video encoding mechanisms have already been developed, e.g., video hashing and video compression algorithms [42] etc.

Modern smart city surveillance cameras are equipped with video coding techniques such as High-efficiency video coding (H.265/HEVC) [40] developed by the ITU, and the visual coding expert group. The HEVC video compression is beneficial for reducing bit-rate, but the quality of the video is also affected [21]. Deep-learning (DL) techniques performed tremendously well in smart city surveillance applications such as remote monitoring [2], transport management systems and road safety management systems [18]. Unlike image segmentation, a semantic segmentation is a process of allocating labels to each pixel of an image in such a way that the pixels with the same label belong to the same characteristics. Utilizing semantic segmentation for S-R extraction can be beneficial for multiple object identification that provides efficient video surveillance [29]. The advancements in smart city surveillance through video analysis not only increases road and traffic security, it also provides monitoring of street crimes. The state-of-the-art DL techniques for semantic segmentation are SegNet [3], U-Net [43], fully convolution network (FCN) [13] and deconvolution network [34]. These techniques can produce accurate semantic segmentation results but often consume high computational cost, high delays, and more time complexity due to the higher number of parameters and depth of layers. In this work we focus on proposing a shallow deep learning based segmentation network i.e. less in parameters, and can extract exact S-Rs without extra region. Moreover, another state of the art approach is proposed in [45] presented a deep learning based traffic video compression in which they are extracting region of interest by DL based localization method. They achieved prominent results for peak signal to noise ratio (PSNR) however, they achieved higher bit-rate because of localization method which unnecessary extra region along with S-Rs.

The main problem for high-efficiency smart city video surveillance encoding is to (1) extract salient-regions efficiently with low inference time. (2) Maintain a trade-off between visual quality and bitrate of the salient regions. Firstly, the existing DL-based S-R segmentation techniques are dense and requires high inference time. Therefore, there is a need of such DL-based network which can extracts salient-regions closer to human visual system with low computational cost and less inference time. Secondly, the video obtained by high-definition smart city surveillance cameras can only be transmitted over networks with higher bandwidths. Therefore, it is mandatory to reduce the video size to transmit it over low bandwidth networks. Traditional encoding techniques such as HEVC, can be used to reduce video size. However, these techniques cannot preserve high-quality of S-Rs while compressing the surveillance video that is why these techniques cannot be directly used for smart city surveillance.

This paper presents an Efficient Shallow Segmentation based Encoding (ESSE) framework which ensures the high quality of salient regions in smart city surveillance video by reducing its size as well. It helps to identify suspected person/vehicle, detect traffic and roads. The scheme uses a less complex solution for deep learning based semantic segmentation. In ESSE, a shallow semantic approach is used for salient region segmentation as it requires fewer layers and parameters that reduces time complexity, which is suitable for surveillance. Moreover, we modified the HEVC encoding based on the extracted S-Rs which helped to reduce video size while preserving quality of salient regions from smart city surveillance video frames. The major contributions of this work are enumerated as follow:

I. We explore the-state-of-the-art techniques by comparing existing tools and evaluating the salient region-based segmentation techniques and video encoding schemes to identify valid problem. We have also identified that the larger video size for high-quality of S-Rs can interrupt the continuous stream of smart city surveillance video in emergency scenarios.
II. A DL-based Shallow Semantic Segmentation Network (S-SSN) is proposed to extract the salient region from input surveillance video frames. The flow of CNN-based layered architecture and the role of the softmax layer is also modified. Although existing literature presents a lot of efforts towards semantic segmentation, to best of our knowledge use of the shallow approach for semantic segmentation in smart city surveillance scenario is not explored much.
III. For efficient smart city surveillance, an efficient segmentation-based encoding framework is proposed and evaluated through case study in smart city environment. We have also performed extensive experiments on varied smart city surveillance videos such as, cross-road, banks, pedestrian in smart city.

The rest of the paper is structured as follows: Sect. 2 presents the literature review for smart city surveillance, S-R segmentation techniques and S-R based HEVC encoding techniques. Section 3 offers the proposed ESSE framework with two phases. In Sect. 4, we discuss the case study results and other analysis. Section 5 concludes our work and provides a few related future research directions.

## Literature review

This section explores the importance of video surveillance in smart cities, the existing schemes for efficient encoding and transmission of surveillance videos in smart cities and then deep learning based salient region segmentation techniques are also explored.

### Video surveillance in smart cities

Video surveillance of smart cities is an important part for development of balanced and secure environment. The first priority video surveillance is to get as much salient information as possible from the surveillance cameras in the shortest time. In [37] Ruben et al., carried out a survey on video surveillance systems current status and future trends. The main significant features and analytics are offered, and also the most general techniques for image/video quality enhancement for surveillance in smart cities. Nemours application of smart cities are included like smart surveillance for transport, social security, services etc. They also discuss most essential emerging deep learning techniques for smart city management and surveillance. Kashif et al., in [24] presented video surveillance for transportation in smart city environment. They have utilized a fuzzy approach to schedule traffic monitoring to avoid traffic congestions in smart cities. However, the authors only focused on traffic monitoring and ignores the pedestrian on roads. Traffic monitoring and guiding system presented in [32] where the authors did extensive study and presented survey for traffic management application in smart city environment. In [30] Saba et al., also opposed vehicular guidance systems based on wireless visual networks for smart city. The authors used graph theory to schedule traffic routes and finding specific locations on map. The proposed system by Saba et al., is efficient for finding shortest route though it is not intelligent enough to accommodate other objects rather than traffic. Another application of smart city surveillance is crowd monitoring presented in [28] where the authors proposed an intelligent computing based framework, the optimization algorithm is applied to compute the feature of crowd motion and measure the correlation between agents based motion model and the crowd data using extended Kalman filtering approach and KL-divergence technique. The experimental results are 96.20% for classifying. The above discussed state-of-the-art studies highlights the need of visual surveillance in smart city environment for managing daily routine services.

### Encoding techniques for smart city surveillance video

In this section, a collection of HEVC coding-based schemes are explored that highlight the S-Rs. HEVC based video coding is used in surveillance for city areas, such as presented in [11]. Kim et al., in [26], was proposed fast CU based HEVC compression for the internet-of-things environment and smart city surveillance. They presented an algorithm, which is based on neighboring block and depth information. In this work, time retention is used as an evaluation matric and achieved a 35% reduction in computational cost. A fast coding unit (CU) depth level selection method is proposed in [4] that can be enhanced along with rate-distortion by extracting regions of interest (RoIs) in smart city surveillance. For RoI selection, a software module is used for background extraction from moving images. The authors also presented a trade-off between PSNR and computational cost for bitrate by using the HEVC rate-distortion feature. In [31], HEVC based rate control technique is explored, which was related smart city visual surveillance and smart video conferencing for better quality RoI encoding by leaving the rest of the image in low quality. In HEVC compression is implemented for moving object segmentation and classification methodology by incorporating HEVC. In this, features from HEVC encoded video were extracted and then used for classification and segmentation for the purpose of video surveillance. The achieved pixel accuracy was 80% for segmentation. RoI-based HEVC compression is presented in [27], where a mechanism for video encoding on social media is proposed using HEVC. It involves a scheduler to select video to encode. They achieved 25% bit-rate reduction. The researchers presented RoI selection based technique by using super-pixel in [41]. They divided pixel regions of an image and assigned priority to the foreground and ignored the background. The achieved accuracy in terms of bit-rate and PSNR is high; however, the RoI extraction by using super-pixel cannot extract salient features semantically. Moreover, Xubien et al. [45] proposed a Perceptual-based Intra Coding Optimization (PICO) algorithm using deep convolution with HEVC. This method is identified that salient region extraction using DN is based on localization, which takes extra regions as well. Therefore, it demands more bit rate to transmit extra regions, that is unsuitable for surveillance.

Our proposed scheme overcomes this issue by utilizing a semantic segmentation approach for salient-region extraction. Moreover, we have identified that applying DCN on classic VGG uses a dense approach with a large number of parameters. It increases computational cost, which is not suitable for the surveillance scenario. In the proposed approach, an improvement has been achieved by reducing both the number of parameters and network depth. The existing video encoding techniques reduces the resolution ratio of surveillance video, which also affects the performance of video analysis tasks, e.g., object/road localization, detection and recognition, vehicle tracking, and crowd monitoring [50]. Therefore, modified S-R based video encoding is more beneficial in video surveillance applications where the high video resolution is required [19].

### Deep learning based salient-region segmentation technique

This section reviews related techniques that use image segmentation to extract salient objects and their regions. Image segmentation is used to extract the S-R in surveillance video frames. The conventional ML image segmentation techniques can be used for salient region extraction. i.e., K-means [10], Density-based spatial clustering of applications with noise (DBSCAN) [15], mean-shift and fuzzy c-means [17]. Hwang et al. [22] proposed multi-object detection and tracking, which focuses on the segmentation of road objects such as vehicles and pedestrians. For deployment, a 3D-lidar is used for sensing the targeted objects on road images, and DBSCAN is utilized to segment those objects for tracking and detection. The achieved pixel-accuracy of segmentation is 67–96% with different time intervals and 3D-lidar frequencies. A pedestrian segmentation and detection scheme using mean-shift segmentation [31] focus on unmanned Ariel vehicles by using a locally collected dataset from surveillance cameras. This scheme was tested on available datasets and achieved pixel-accuracy of 76% calculated by (1) where, ${t}_{i}$ is the total number of pixels belonging to class $I$, and ${p}_{ii}$ represents the number of true positives. Above discussed ML-based segmentation techniques have low time complexity; however, they are sensitive to noise and require pre-processed data such as pixel intensity information and extracted edges in images. These computer vision and ML state-of-the-art algorithms have constraints in their ability to segment natural images with respect to the object classification. The input dataset with many levels of abstraction can also involve major manual-tuning for all images explicitly.

1 $$Pixel\_level\_Accuracy={\sum}_i{p}_{ii}/{\sum}_i{t}_i$$

The state-of-the-art DL techniques for semantic segmentation include SegNet [3], U-Net [36], fully convolution network (FCN) [39] and deconvolution network [34]. These techniques can produce accurate semantic segmentation results but often consume high computational cost, high delays, and more time complexity due to the higher number of parameters and depth of layers. SegNet, U-Net, deconvolution network comprises of 26, 23, and 39 convolutional layers. These DL-based networks have a higher number of parameters which delays the inference that may affect the performance of a video surveillance encoding system. In [38] Cristiano et al., presented a region of interest (RoI) based video encoding for video conferencing application. They have used viola and jones [47] algorithm to determinate the RoIs and propose a rate control scheme that allows using large bitrates in RoIs to achieve high image quality in such areas and imposes bitrate limitations in the remaining regions. DL consists of ample techniques, which automatically learn features from raw input data itself. DL-based image segmentation is referred as semantic segmentation. Semantic segmentation is the process of associating each pixel of an image to a selected output class label, i.e., building, roads, trees, cars, etc. Pixel-accuracy of semantic segmentation is formulated as explicit labeling of each pixel where Xi ∈ R3 all three channels of an image corresponding to a label Yi from a static set Ψ. For N number of observations, X = {X_1_,…,X_N_}a set of labels Y = {Y_1_,…,Y_N_} is predicted taking values in Ψ_N_. Pixel-level accuracy is used as evaluation matrix for semantic segmentation. Accordingly, these evaluation matrices use a confusion matrix C of pixel level, which sums inferences for all the images of dataset as given in (2) [27].

2 $${C}_{ij}=\Sigma\ I\in D\mid \left\{Z\in I\ s.t.{SI}_{gt}(Z)=i\&{SI}_{ps}(Z)=j\right\}$$

In Eq. (2), SIgt (Z) is the ground truth of pixel Z in I image, SIps is the predicted class based on labels, and |A| carries the elements of the set A. In detail, the Cij is the total numbers of pixels belongs to ground truth label i predicted as j. Some of the popular deep convolutional semantic segmentation networks based on convolutional neural networks (CNNs) are discussed below. In [41], a FCN for semantic segmentation based on VGGNet [50], GoogleNet [19] and ResNet [10] is presented. The FCN combines features by using fully convolutional layers and achieved 89.19% of accuracy, and 62.3% mean intersection over union (mean IoU) on PASCAL VOC [15] dataset. However, the FCN computational cost is high due to the fully connected layers. Undoubtedly, methods like the above have been in use to solve various issues, such as to enhance visual quality. Their performance is based on the attention mechanism, that prominent texture regions might attract more attention than sparse texture regions. Moreover, they can be used for special needs and improve the visual experience. However, most of these algorithms are based on texture or color-features to identify the salient regions from an image. Saliency extraction based on deep learning is rarely explored, which is closer to the human perception system and also suitable for a surveillance scenario. a brief review of state-of-the-art techniques are listed in Table 1

**Table 1:** State-of-the-art techniques

Author and References	Dataset	Technique(s)	Results/accuracy	Weakness/remarks
Kumar et al. [30]	Own Extracted dataset	Kalman filtering and KL-divergence technique	96.20% (classification)	Limited feature (only traffic) The utilized data is not publically available. No other objects on road are utilized to monitor
Hwang et al. [4]	Kitti	DBSCAN	67%-96% (pixel accuracy)	Challenging for different weather/light conditions
[31]		Mean-shift segmentation	76% (pixel accuracy)	Works for specifically pedestrians
Badrinarayananet al. [3]	CamVid	SegNet	50.02 mIoU	Consume high computational cost due to extensive depth of layers
Ronneberger et al. [43]	ISBI cell tracking dataset	U-Net	92.03% (pixel accuracy) 77.56% mIoU	Results in high delays due to the higher number of parameters Designed for binary class segmentation
Hyeonwoo et al. [34]	PASCAL VOC 2012	Deconvolution network	69.6 mIoU	Need more time for inference due to a dense network
Jonathan et al. [13]	SIFT flow	Fully convolutional network	85.2% (pixel accuracy)	Requires features from previously learned networks which makes it unsuitable for road security scenarios

## Proposed efficient shallow segmentation-based encoding framework for smart city surveillance

**Fig. 1:**
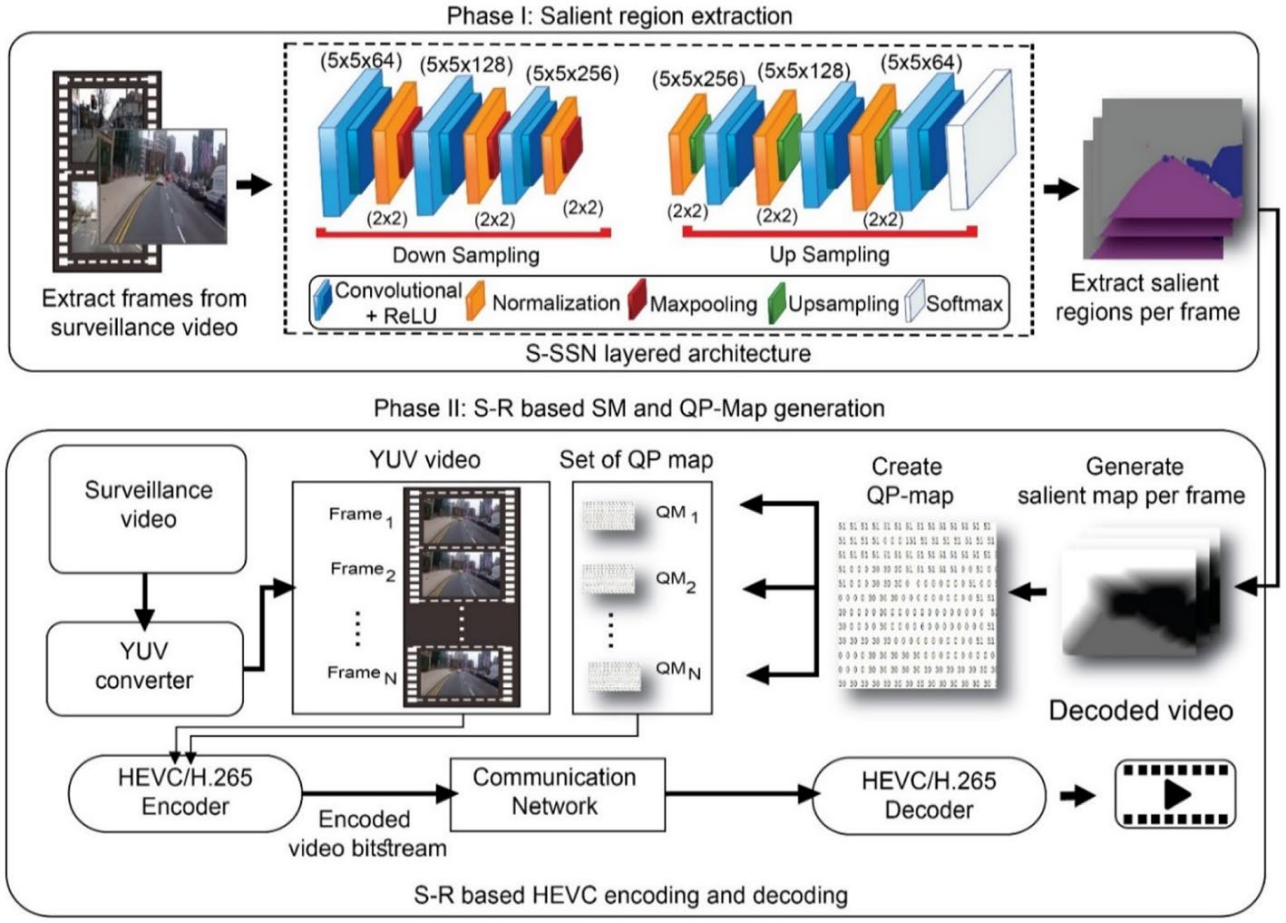
Proposed ESSE Phases for S-R extraction, QP-Map generation, and HEVC encoding–decoding

This section presents the Efficient Shallow Segmentation-based Encoding (ESSE) framework for smart city video surveillance of vehicular traffic, roads and human beings. It demands a high quality semantic segmentation technique to extract the salient regions like humans and vehicles where video size is also reduced to ensure uninterrupted transmission of surveillance video especially for emergency scenarios. For bulk video analysis the data should be light weight to prevent storage and quality issue. In this work, we present a framework for video efficient surveillance video transmission for targeted visual analysis based on salient video encoding which helps to attain high quality for targeted salient region with low file size. The proposed framework could help to segment salient region such as humans and vehicles of each type from video frame and transmit in low bandwidth capacity. The proposed framework can help in intelligent transport management, street crime, and road hazards. The proposed framework is divided into two phases as shown in Fig. 1.

In Phase I, the salient region (S-R) is segmented from the input video frames by using our proposed Shallow Semantic Segmentation Network (S-SSN) which is trained using Mapillary vistas dataset divided into training data (18,000 images) and testing data (2000 images). For training the parameters are set such as ${\varvec{b}}{\varvec{a}}{\varvec{t}}{\varvec{c}}{\varvec{h}}\_{\varvec{s}}{\varvec{i}}{\varvec{z}}{\varvec{e}}=\boldsymbol{ }6$, ${\varvec{n}}{\varvec{u}}{\varvec{m}}{\varvec{b}}{\varvec{e}}{\varvec{r}}\_{\varvec{o}}{\varvec{f}}\_{\varvec{e}}{\varvec{p}}{\varvec{o}}{\varvec{c}}{\varvec{h}}=75$, ${\varvec{n}}{\varvec{u}}{\varvec{m}}{\varvec{b}}{\varvec{e}}{\varvec{r}}\_{\varvec{o}}{\varvec{f}}\_{\varvec{c}}{\varvec{l}}{\varvec{a}}{\varvec{s}}{\varvec{s}}{\varvec{e}}{\varvec{s}}\boldsymbol{ }=\boldsymbol{ }5$. The S-R is extracted for each frame of input surveillance video. We identified that the existing state-of-the-art DL-based techniques involve a dense layer pattern which requires more computational cost, processing time and less accuracy. We have designed and evaluated a large number of layer patterns and finally the proposed model named S-SSN is proved to be the best suitable with less computational cost and better accuracy. Proposed model includes less number of layers, therefore, we call it a shallow network instead of dense. Existing approaches including U-net, DN and SegNet are dense with more number of parameters and less accuracy and mIoU. These are not suitable for visual analysis for time critical emergency system like road surveillance.

In phase II, the extracted frames are preprocessed to generate a set of the salient map (SM) for each S-R per frame. Next, the SM is further utilized to create QP maps for all extracted N frames. Furthermore, a set of QP maps is also maintained for all N frames. Meanwhile, YUV frames are also created from the original input video. Next, the modified HEVC encoder takes each QP map from the set to enhance the QP values of each YUV frame. In this case, we modified the default HEVC by considering the QP values to sustain the quality for salient regions like the vehicles and pedestrians in the frame. We reduce the quality of irrelevant areas by increasing QP values that result in reducing the overall size of the video. Modified HEVC encoder generates the encoded video bit-stream with a reduced size that requires less bandwidth. It enables the continuous the remote monitoring of ambulance through surveillance camera and the video can be transmitted even when bandwidth is low. This proposed scheme can facilitate remote visual analysis by providing high quality for S-Rs to analyze the data at cloud. It also reduces the video size that enhances the chances of uninterrupted video sharing even if bandwidth is limited. A list of notations is presented in Table 2.

**Table 2:** List of notations

Sr	Notation	Description
1	${\mathrm{t}}_{\mathrm{i}}$	Total number of pixel belongs to a class
2	$\mathrm{r},\mathrm{ c}$	Row index of a frame
3	frame	A 2D image from the video with r rows and c columns
4	Video_Frames	Number of frames in an input video
5	fn	Index for frame starting from the first frame
6	SM[]	An array of Salient_Map containing frames with S-R
7	N	Number of Video Frames
8	wd	Diagonal weight
9	wn	Neighbor weight
10	QM_1_…_N_	QP map for 1…N number of frames
11	QP	Quantization parameter of HEVC
12	L_1_–L_N_	Layers for proposed S-SSN
13	i	S-SSN Layer index
14	${\mathrm{a}}^{\left(\mathfrak{i}\right)}$	Activation function on each S-SSN layer
15	${\mathrm{l}}^{\left(\mathfrak{i}\right)}$	Upsampling stage for S-SSN layers

### Phase-I: salient region extraction

The proposed S-SSN is a DL-based segmentation network that is based on a shallow semantic approach. In the approach, a shallow architecture is used for the segmentation network instead of dense architecture. This is because shallow architecture has less time complexity due to fewer parameters, and therefore, it suits surveillance scenarios. The S-SSN semantically extracts those classes which are important for road surveillance, i.e., vehicles, motorbike, pedestrian, and roads. The dataset used during training and testing phase of proposed S-SSN is open-source Mapillary Vistas and Camvid, which is considered to be a benchmark [8]. It comprises of 18,000 training and 2000 validation images. It contains 66 instance-specific object categories. It covers all North and South America, Europe, Africa, Asia, and Oceania geographic locations. It contains high weather variations, i.e., sunny, rainy, snowing, fog, and haze. It has been captured during different times like dawn, daylight, dusk, and night. Different camera sensors are used to capture this dataset, which varies in focal length, aspect ratio, and camera noises. For the dataset, a variety of capturing viewpoints have been used, like capturing from the road, sidewalks, and off-road. Our selected experimental dataset covers major variations of the environment to ensure robust S-R segmentation. The dataset also includes extra-ordinary weather and environment variations. The S-SSN is trained on the Mapillary vistas and Camvid benchmark road dataset separately with a ratio of 80% for training, and 20% is for testing. After training, the S-SSN collects a set of feature weights and uses them for prediction in testing phase. Figure 1 illustrates that video frames are extracted from surveillance video and then fed into S-SSN layered architecture for salient region extraction. We also improved the order of layers for up-sampling and down-sampling to improve efficiency of this module. During down-sampling, the layers L_1_–L_9_ from left to right take the frame as input and then down-sample it.

During up-sampling, layers start from L10–L17, where the output image of size 28 × 28 × 256 from down-sampling is fed into the network as input. The input image is up-sampled by using the convolution layer, which comprises of 256, 128, and 64 filters of size 5 × 5. The up-sampling and normalization layers return an image of size 224 × 224 × 5, where the 224 × 224 is height and width of image and 5 are the number of classes for segmented salient regions, i.e., vehicle, pedestrian, road, bicycle/bike, and the background. In the end, S-SSN uses a softmax layer that calculates the probability of each class in contrast to all possible numbers of classes. The S-SSN generated an extracted salient region frame for N number of frames, i.e. $\{{frame}_{1},{ frame}_{2}, \dots ,{ frame}_{N}\}$. In the overall process, S-SSN takes an input video stream ${vid}_{input}$ having $n$ frames $\mathfrak{x}$ such that ${vid}_{input}=\left\{{\mathfrak{x}}_{1}, {\mathfrak{x}}_{2},\dots , {\mathfrak{x}}_{n}\right\}$ where each frame is a three-channel image with height $h$ and width $w$ such that: ${\mathfrak{x}}_{n}\in {\left[0;4\right]}^{h\times w\times 3}$, the objective is to learn a function $f$ that produces multi-class masks ${\mathbb{y}}\in {\left[0;4\right]}^{h\times w\times 3}$ for ground-truth or salient region ${S-R}^{gt}$ where $r$ represents the row, and $c$ represents the column of ${\mathbb{y}}$ label as given in (3).
3

S-SSN involves the CNN layers, including convolution layers, max-pooling layers, up-sampling layers, ReLU, and batch-normalization. All layers are stacked to calculate activation function at ${\mathfrak{i}}{\rm th}$ hidden-layer of the network at ${k}{\rm th}$ stage as given in (4) where ${a}^{\left(\mathfrak{i}-1\right)}$ represents the input to the layer, ${\mathbb{w}}^{\left(\mathfrak{i}\right)}$ and ${\mathcal{b}}^{\left(\mathfrak{i}\right)}$ are weights and biases of the ${\mathfrak{i}}{\rm th}$ layer, respectively. The semantic segmentation network involves three components, including polling, up-sampling, and softmax. The pooling and up-sampling of the features are expressed in (4) and (5) at every stage ${l}^{\left(\mathfrak{i}\right)}$:
45

The softmax stacks these layers in a particular order, as expressed in (6). In this scenario, each pixel of the input $\mathfrak{x}$ has given a representative class calculated on the ground of $softmax,$ where $k$ represents the down-sampling stage, and $l$ represents the up-sampling stage.
6

### Phase-II: S-R based SM and QP map generation for HEVC encoding

In Phase-II, we created SM to generate QP maps. These maps are further used for video encoding using HEVC, which is discussed in the following sub-sections.

#### S-R based salient map creation

The frames with extracted salient regions are preprocessed to enhance the visibility and area around S-R. Next, a for each S-R is generated using distance transform [7]. In Algorithm 1 for SM creation, a set of video frames with extracted S-R are used as input. Step 1 takes the first frame for initialization, and step 2 sets a count for all number of frames to N. In step 3, an array is set for SM, and step 4 iterates frames until it lasts whereas step 5 iterate N numbers of frames. Step 6 is to apply forward pass on the extracted frame. In step 7, iterations are applied for rows where row index r ranges from {2, 3, …, *m* − 1}, and m represents rows count in a frame. Step 8 explores the iterations for the number of columns where column index c ranges from {2, 3, …, *n* − 1}, and n is the number of columns in the frame. In step 9, the forward pass is calculated by moving diagonally on the extracted frame where wn and wd are diagonal weights in neighbors which are used to calculate distance from each pixel location of the frame. During backward pass on the video frame, step 13 shows iterative repetition in reverse order for rows index r ranging from {m − 1, m − 2, …, 2}. Similarly, column index c is iterated in a reverse manner ranging from {n − 1, n − 2, …, 2}, as shown in step 14. In step 15, the backward pass moves diagonally in backward direction to calculate the distance of each pixel from its location by using $wn$ and $wd$. Next, SM is created for each extracted frame, as illustrated in step 18.

#### QP map generation

**Fig. 2:**
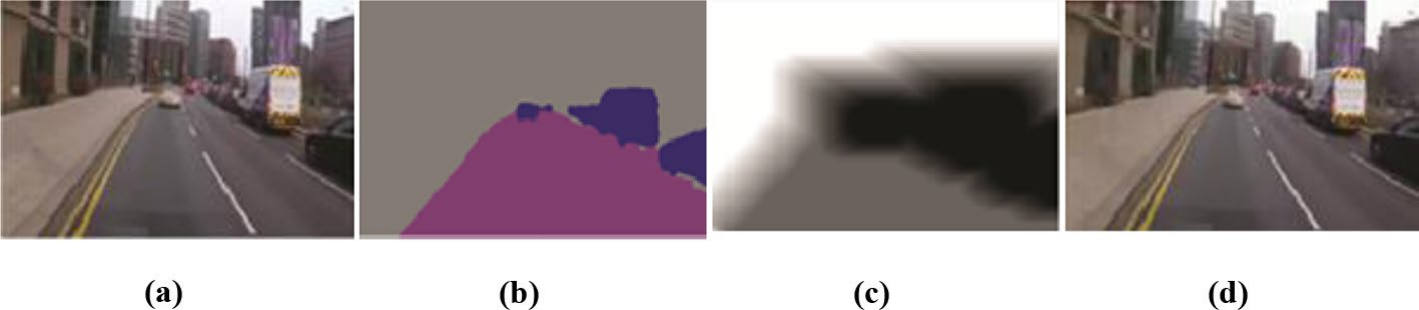
S-SSN and modified HEVC for **a** input frame **b** S-Rs, **c** SM and QP Map and **d** encoded frame

SM is used to create a QP map, which is further utilized to adjust the quality of S-Rs and non-SRs. The set of SM creates a set of QP maps for N frames. QP maps $\{{\mathrm{QM}}_{1}, {\mathrm{QM}}_{2},\dots , {\mathrm{ QM}}_{\mathrm{N}}\}$ corresponds to $\{{\mathrm{fn}}_{1}, {\mathrm{fn}}_{2},\dots , {\mathrm{fn}}_{\mathrm{N}}\}.$ For an evaluation scenario, a surveillance video is converted into YUV format to feed as input in modified HEVC with S-R based QP map. After that, the modified HEVC encodes the surveillance video by using S-R based QP map to reduce the quality of non-SRs and enhance the quality of the desired region like vehicles, roads, and motorbikes. As illustrated in Fig. 2, an S-R based QP-map is created from video by identifying S-Rs and SM.
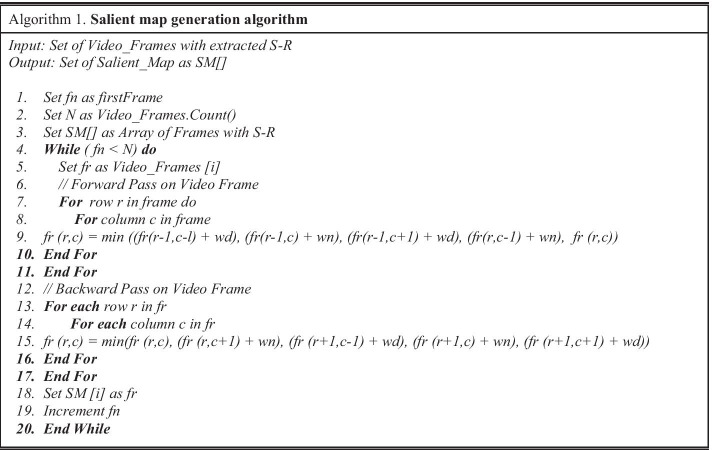

#### Surveillance video encoding

We have utilized default HEVC reference test model HM 16 [20] for modifications. In the default HEVC, the entire frame is encoded to reduce its size and quality, but it also results in reducing the quality of S-Rs. We have enhanced the functionality of default HEVC by incorporating S-Rs based quality adjustment during encoding. QP maps play a vital role in enhancing the quality of S-Rs, including roads and vehicles, but reducing the quality of background to reduce overall video size. The modified HEVC uses the QP map based on S-R locations instead of using default predicted QP values. HEVC involves 2-D transforms [44], scaling and quantization [46] module. It takes a QP map as input and also takes the input video. The output of this module is fed into context-adaptive binary arithmetic coding (CABAC) that generates encoded bit-stream as shown in Fig. 2. HEVC divides video frames into equal coding tree units (CTUs), which are equally sized blocks. The range of CTU size varies from 16 × 16 to 64 × 64 with two chroma (Cb and Cr), one luma (Y), and coding tree blocks. Modified HEVC receives input YUV video and corresponding QP maps to perform salient region-based quality control. This encoded video is transmitted towards users who can decode it. In this scenario, CTUs in high priority S-Rs are encoded using low QP values and vice versa. The outcome of S-SSN segmentation and modified HEVC is presented in Fig. 2, where SM and QP map are generated on the basis of prioritized S-R like vehicles, pedestrians, and motorbikes. In this scenario, the base QP value is 20 for S-Rs with good quality and 51 for non-SRs to blur its background region by preserving saliency along with reduction in video size.

## Result and analysis

**Fig. 3:**
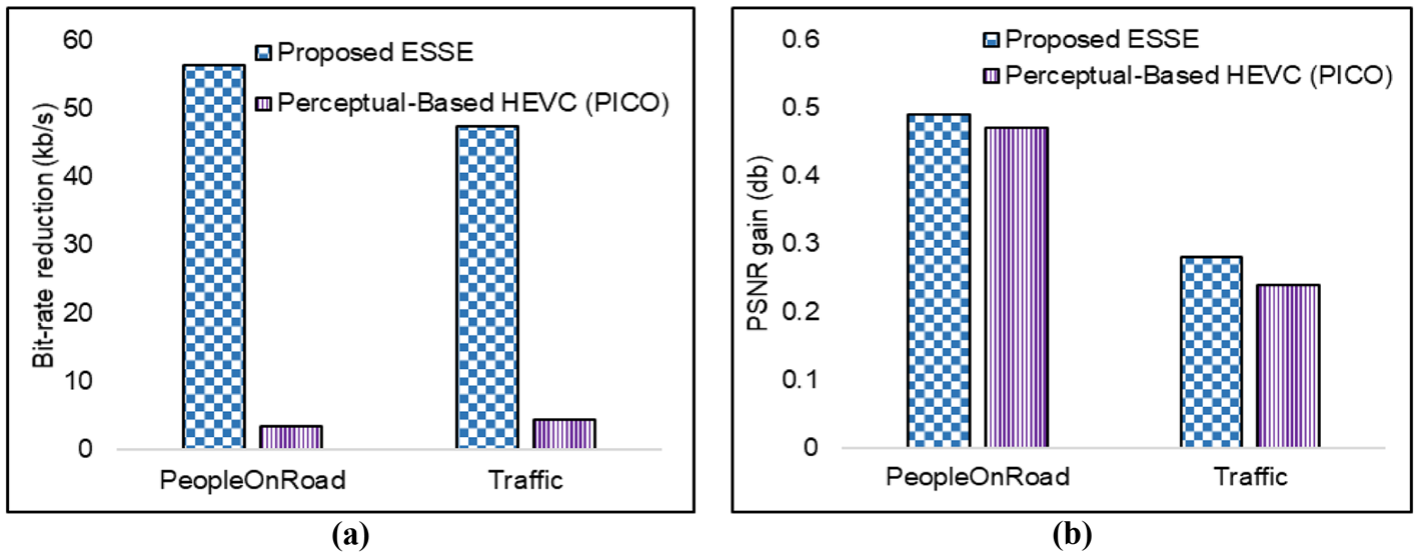
**a** Bit-rates are shown for comparison with PICO **b** PSNR gain of proposed ESSE

**Fig. 4:**
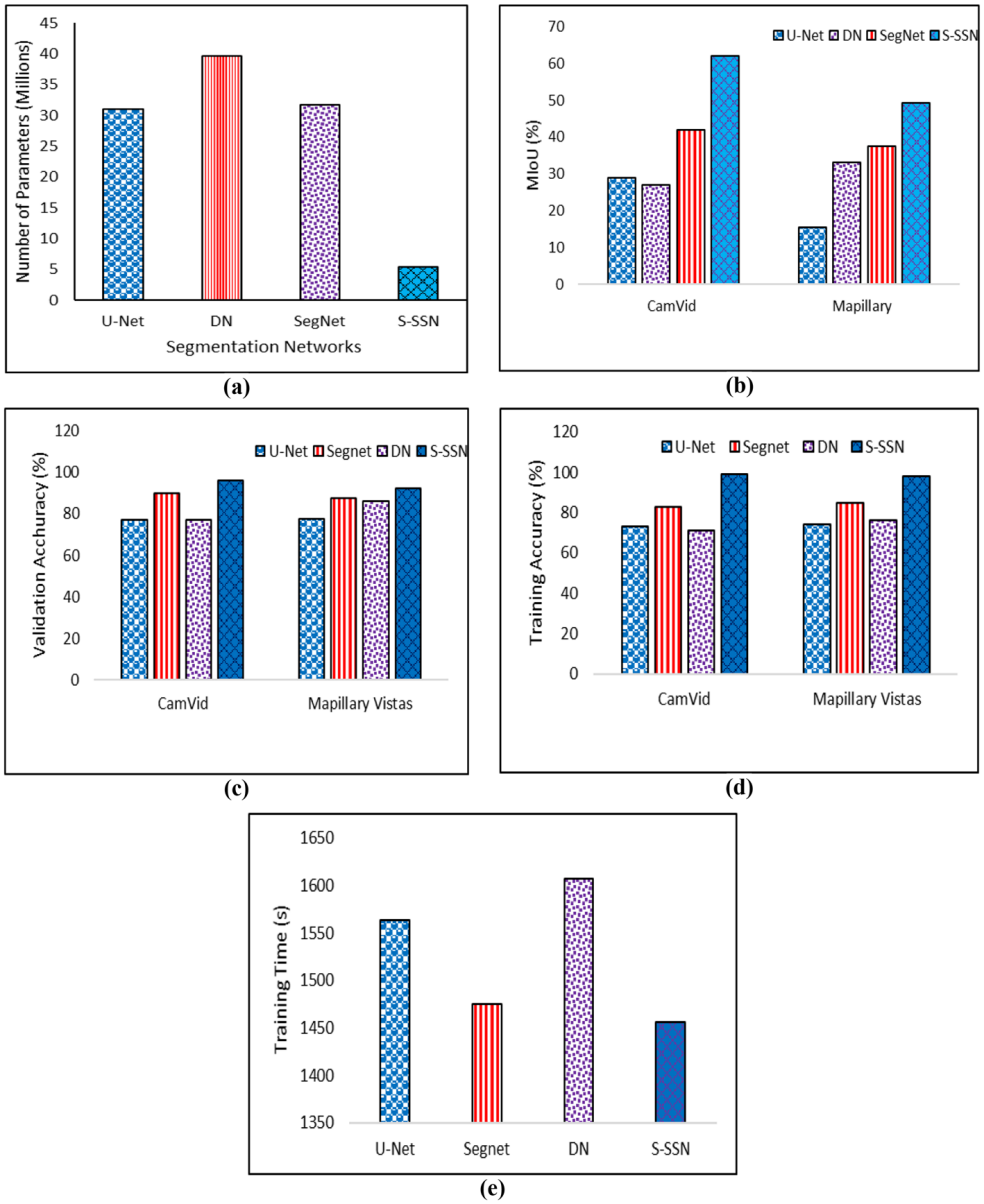
For segmentation networks, the number of parameters are shown in (**a**) whereas mean IoU are shown in (**b**), (**c**) and (**d**) carries the validation and training accuracies. **e** Average time for training segmentation networks

**Fig. 5:**
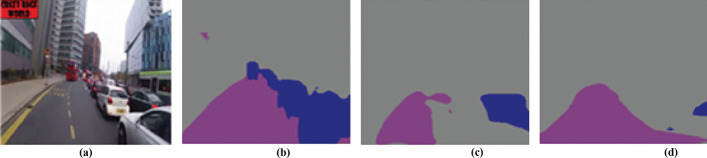
Visual segmented results for **b** S-SSN **c** SegNet **d** DN **a** is taken from tested video

**Fig. 6:**
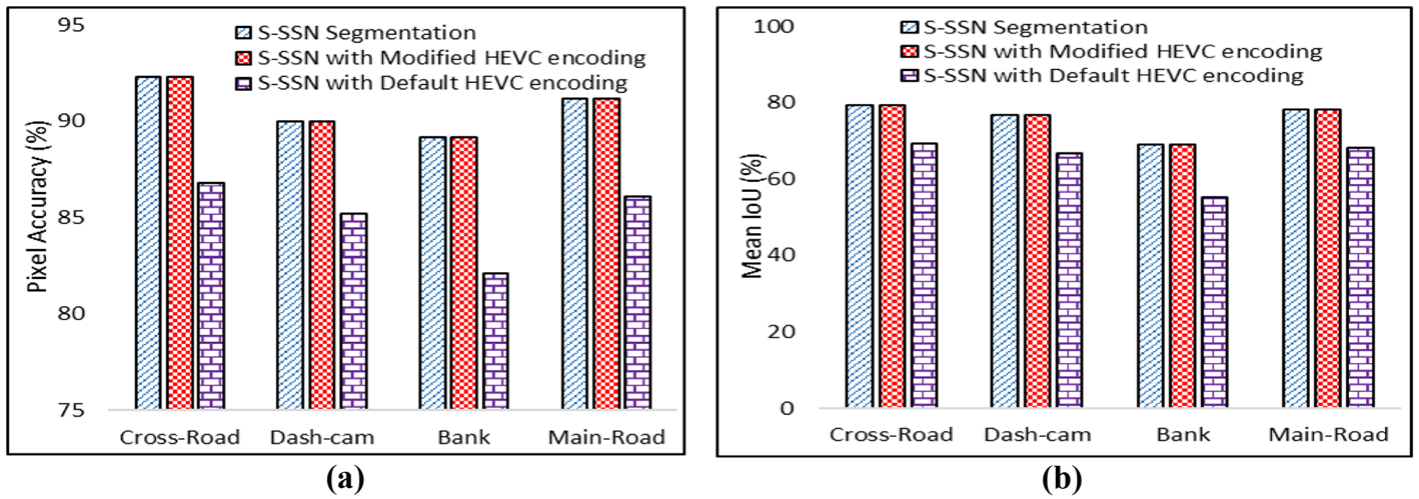
For different video sequences, the Pixel accuracy and Mean IoU are presented in (**a**) and (**b**)

This Section explores the performance of the implemented ESSE scheme as compared to its counterparts. For the experimental setup, the Adadelta optimizer is used for training the proposed S-SSN with default parameters by using Keras and Tensorflow python libraries. All experiments are performed on the Google Colab platform with Nvidia Tesla K80 GPU having 11 GB VRAM and 12 Gb of RAM [25]. HEVC reference test model HM 16 [16] is used. For encoding the surveillance video frames as per the generated salient-map, the default available HEVC test model is used. For a fair comparison, we have established the same experimental setup for other state-of-the-art deep learning based techniques. We focused on four classes including pedestrians, vehicles, motorbike/bicycle, and road. To evaluate the performance of proposed ESSE framework more extensive experiments are conducted and the results are evaluated for bit-rate, peak signal to noise ratio, number of parameters for segmentation networks, mean intersection over union (Mean IoU), pixel accuracy, PSNR db, and bit-rate percentage. We video encoding comparison we have compared ESSE framework with PICO [46] that applies deep learning by using two test video sequences “PeopleOnRoad” and “Traffic” from JCT-VC [6] in Fig. 3a, b. Next, we compared with the state-of-the-art segmentation methods, including SegNet [43], U-Net [13], and deconvolution network (DN) [45] in Fig. 4a-e. Furthermore, Fig. 5a-d presents visual results of segmentation for DL based segmentation networks. The comparison of different environments video sequences such ad Main-road [12], Cross-road [23], Bank [49], and Dash-cam [33] is presented in Fig. 6a, b.

### Bit-rate for video transmission

It is quite essential to measure the bit-rate due to reduction in the size of encoded video to evaluate the surveillance video on low bandwidth networks. We use the same evaluation matrices as in [45] by considering full-frame and S-R as well. We use three quantization settings where the QP value ranges displayed in Table 3 where 0–20 is for high-quality video encoding, 25–35 for medium quality, and 40–51 for low quality. During the experiment, the same regions of frames are utilized. Table 3 elucidates that proposed ESSE dominates by consuming less bit-rate of 2552.19 kb/s for QP value 20 with high-quality video whereas default HEVC consumes more up to 5939.27 kb/s. To calculate the decreased value of bit-rate, Bjontegaard delta bit-rate (BDBR) [6] is used. The rate-distortion (RD) curves of proposed S-R encoding and default HEVC encoding is utilized to calculate BDBR. ESSE attained a BDBR decrease of 56.92%, 52.03%, and 25.87% for high-quality, medium quality, and low-quality settings for QP values, respectively. Furthermore, Table 4 elucidates that bit-rate for default HEVC is 2256.851 kb/s, 5939.277 kb/s, 4939.265 kb/s, and 3256.762 kb/s for cross-road [16], dash-cam [9], main road [1] and bank [25] video sequence, respectively. For a similar sequence, ESSE framework consumes less bitrate as 774.339 kb/s, 2552.192 kb/s, 1952.183 kb/s, and 1274.345, respectively. The gain in bit-rate reduction from Fig. 3a show that bit-rate reduction for video sequences like “PeopleOnRoad” and traffic scenario is 3.38% and 4.4% for PICO [45], respectively. For the same sequence, proposed ESSE dominates by reducing the bit-rate by 56.51% and 47.49%, respectively. The difference of obtained values for bitrate is calculated as shown in (7) [45]. Results show that the proposed solution has a trade-off between bandwidth resources and visual quality of S-R in smart city surveillance videos to achieve better bit-rates.

**Table 3:** BD-PSNR and BDBR results for ESSE framework and default HEVC for tested surveillance video

QP	Default HEVC bitrate (kb/s)	Proposed ESSE bit-rate (kb/s)	Avg Δ BD-BR (%)	Avg PSNR in S-R		Avg Δ BD-PSNR(dB)	Avg PSNR of Frame in dB		
				Default HEVC	Proposed ESSE		Default HEVC	Proposed ESSE	
*High-quality setting*									
0	44,319.73	16,865.82	− 56.92	67.43	67.56	5.35	64.4205		60.2447
5	29,600.50	13,299.33		59.80	59.91		58.1584		53.2501
10	17,587.23	7204.11		56.11	56.25		54.0684		50.7699
15	10,377.49	4312.31		52.71	52.84		50.8862		47.3834
20	5939.27	2552.19		49.61	49.77		47.9496		42.1739
Medium-quality setting									
25	3506.91	1525.07	− 52.03	46.47	46.75	4.23	44.9181		39.4887
30	2028.57	960.64		43.40	43.29		41.9901		36.7874
35	1120.18	556.39		40.26	40.45		38.9366		33.1159
Low-quality setting									
40	610.03	320.29	− 25.87	37.15	37.00	1.18	35.9031		31.1556
45	319.66	216.45		34.02	33.50		32.8828		30.5697
51	136.70	136.70		30.50	30.50		29.4725		29.4725

**Table 4:** Bit-rate savings of ESSE for different smart city surveillance videos encoded at base QP value of 20

Surveillance videos	Default HM bit-rate (kb/s)	ESSE bit-rate (kb/s)	Bit-rate savings (%)	Default HEVC PSNR (db)	ESSE PSNR (db)
Cross-road [16]	2256.851	774.339	65.68	45.15	45.18
Dash-cam [9]	5939.277	2552.192	57.02	46.94	46.95
Main-road [1]	4939.265	1952.183	60.47	46.61	46.62
Bank [25]	3256.762	1274.345	60.87	44.86	44.89

7 $$\Delta BR={BitRate}_{proposed}-\frac{{BitRate}_{HM}}{{BitRate}_{HM}}100$$

### Peak signal to noise ratio

We calculated the PSNR value of the complete frame to analyze a fair comparison of ESSE framework with default HEVC. The average PSNR value of default HEVC for the complete frame is higher than ESSE since the proposed ESSE focuses on preserving the S-R while maintaining the low bit-rate by assigning higher QP values to the regions which are non-S-Rs. Therefore, the proposed ESSE achieved the higher PSNR value. The results from Table 4 presents a comparison of PSNR for full-frame and S-R as well in case of default HEVC and proposed ESSE framework. In the case of S-R with a baseline QP value of 20, ESSE achieved the PSNR of 49.77 dB with a bit-rate of 2552.19 kb/s, whereas default HEVC obtains 49.61 dB with low bit-rate of 5939.27 kb/s. In the case of full-frame comparison, ESSE achieves 47.95 dB whereas default HEVC obtains 42.17 dB. Moreover, the proposed ESSE is compared with state-of-the-art deep learning-based PICO [45] by utilizing two test video sequences “PeopleOnRoad” and “Traffic” from JCT-VC [8]. We make a fair comparison between perceptual based HEVC or PICO [45] and proposed ESSE with modified HEVC. We first processed the two video sequences with ESSE and then calculated the difference of obtained values for PSNR by using the Eq. (8) from [45]. To quantify the gain in the visual quality of the surveillance video, we have utilized the Bjontegaard delta peak signal-to-noise ratio (BD-PSNR) [6]. It is observed that BD-PSNR is improved by 5.35 dB, 4.23 dB and 1.18 dB for high, medium, and low-quality settings for QP values. Table 4 elucidates the PSNR values for other smart city environment video sequences, where the default HEVC achieved PSNR as 45.15 db, 46.94 db, 46.61 db, 44.84 db for cross-road [16], bank [25], main road [1] and dash-cam [9], respectively. For the same video sequences, the proposed ESSE achieved the PSNR as 45.18 db, 46.95 db, 46.62 db and 44.89 db, respectively. The achieved PSNR value of ESSE and Default HEVC is almost same which shows that the proposed ESSE framework achieve same quality video as HEVC but ESSE achieves better quality for salient regions with lower bit-rates. Figure 3b represents the gain in PSNR in db. For same experimental conditions the proposed ESSE framework achieved 0.49 db gain in PSNR for PeopleOnRoad and 0.28 db gain for “Traffic” video sequence whereas PICO [45] obtained 0.47 db and 0.24 db, respectively.

8 $$\Delta PSNR={PSNR}_{Proposed}-{PSNR}_{HM}$$

### S-R segmentation accuracy

A quantitative measure is to check the accuracy of S-R segmentation and compare it with state-of-the-art semantic segmentation networks, including SegNet [3], U-Net [36], and DN [34]. Pixel-level accuracy evaluates percentage for correctly segmented pixels calculated as $\sum_{\mathrm{i}}{\mathrm{p}}_{\mathrm{ii}}/\sum_{\mathrm{i}}{\mathrm{t}}_{\mathrm{i}}$. The term ${\mathrm{p}}_{\mathrm{ii}}$ represents the number of true positives, ${\mathrm{t}}_{\mathrm{i}}$ represents the number of pixels, i belongs to class $i$ and ${\mathrm{p}}_{\mathrm{cl}}$ shows the total number of classes. The ${\mathrm{p}}_{\mathrm{ij}}$ depicts the pixels from class $i$ which are predicted and belongs to class j, whereas ${\mathrm{p}}_{\mathrm{ji}}$ is the wrongly rejected number of pixels for class i. The mean IoU [39] is the percentage of correctly overlapped pixels between the ground truth label and segmented output calculated as $(1/{\mathrm{p}}_{\mathrm{cl}})\sum_{\mathrm{i}}{\mathrm{p}}_{\mathrm{ii}}/({\mathrm{t}}_{\mathrm{i}}+ \sum_{\mathrm{i}}{\mathrm{p}}_{\mathrm{ji}}-{\mathrm{p}}_{\mathrm{ii}})$. Figure 4a elucidates the number of parameters utilized in the network where proposed S-SSN dominated by utilizing only 5.46 million parameters whereas U-Net, DN, and SegNet utilized 31.03 million, 39.64 million and 31.71 million, respectively. The proposed S-SSN in Fig. 4b obtained mIoU of 49.26% and 62% for Maplillary vistas and CamVid dataset. The other state-of-the-art techniques for Mapillary vistas obtained mIoU of 15.51%, 33.16% and 37.6% for U-Net, SegNet, and DN. For CamVid mIoU the other compared techniques achieved 29%, 27%, and 42% for U-Net, SegNet, and DN.

Figure 4c presents pixel-level validation accuracy for Camvid dataset of 96%, whereas for the same experimental settings, the U-Net, DN, and SegNet achieved 77%, 77%, 90%, respectively. It also explores that proposed S-SSN achieved the highest mean pixel level validation accuracy of 92% as compared to 77%, 86%, and 87 for U-Net, DN, and SegNet, respectively for Mapillary vistas dataset. Figure 4d illustrates the pixel-level training accuracy for Mapillary vistas and Camvid datasets for comparative state-of-the-art segmentation networks. Results show that the proposed S-SSN model predicts the S-Rs from Camvid dataset with pixel-level training accuracy of 99% whereas for the same experimental settings, the U-Net, DN, and SegNet achieved 73%, 71%, 83%, respectively. It also explores that proposed S-SSN achieved the highest training accuracy of 98% as compared to 74%, 85%, and 76%for U-Net, DN, and SegNet, respectively for Mapillary vistas dataset. Furthermore, Fig. 4e illustrates time consumed for training of S-SSN and other compared state-of-the-art techniques. The S-SSN consumed 1563 s whereas the U-Net, SegNet, and DN consumed 1475 s, 1607 s and 1456 s. The above mentioned comparative results are calculated after deep analysis. It can be observed from the above graphs that the proposed S-SSN is best suitable for road surveillance scenarios in terms of validation accuracy, mIoU, time and number of parameters.

Figure 5 shows the visual segmented result as compared to state-of-the-art semantic segmentation networks. Figure 5a represents the original frame from the surveillance video sequence [12], whereas Fig. 5b shows segmented results of the proposed S-SSN model. Figure 5c and d shows results of segmentation by SegNet and DN. Results demonstrate that S-SSN achieves significant segmentation results for S-R and is a better S-R extractor for surveillance videos as it requires fewer computations due to the shallow approach as compared to counterparts. Figure 6a elucidates the pixel-level accuracy percentage when results are obtained for four video sequences. Results show that simple S-SSN segmentation, and S-SSN segmentation with modified HEVC achieve 92.31%, 90.02%, 89.18%, 91.2% pixel accuracy for cross-road [16], dash-cam [9], bank [25] and main road [1] video sequence, respectively. Values for S-SSN segmentation and S-SSN with modified HEVC are same because it shows that pixels with S-SSN segmentation sustain higher pixel accuracy even after performing modified HEVC encoding. On the contrary, the default HEVC encoding reduces the pixel accuracy as 86.78%, 85.17%, 82.11%, and 86.1% for the same video sequences with same S-SSN as well. Figure 6b illustrates Mean IoU as 79.31%, 76.78%, 69.05%, 78.06% for S-SSN segmentation, and S-SSN with modified HEVC for cross-road [16], dash-cam [9], bank [25] and main road [1] video sequences.

## Case study results in smart city scenario

This Section presents case study test scenarios results for our proposed ESSE framework for smart city video surveillance on baseline QP of 20 for S-Rs. We have conducted extensive experiments in different environments and luminance conditions for vehicles, pedestrians, motorbike and bicycles to evaluate the performance of our proposed ESSE framework. Case studies are as follow:

### Case study 1: Identification of pedestrian

**Fig. 7:**
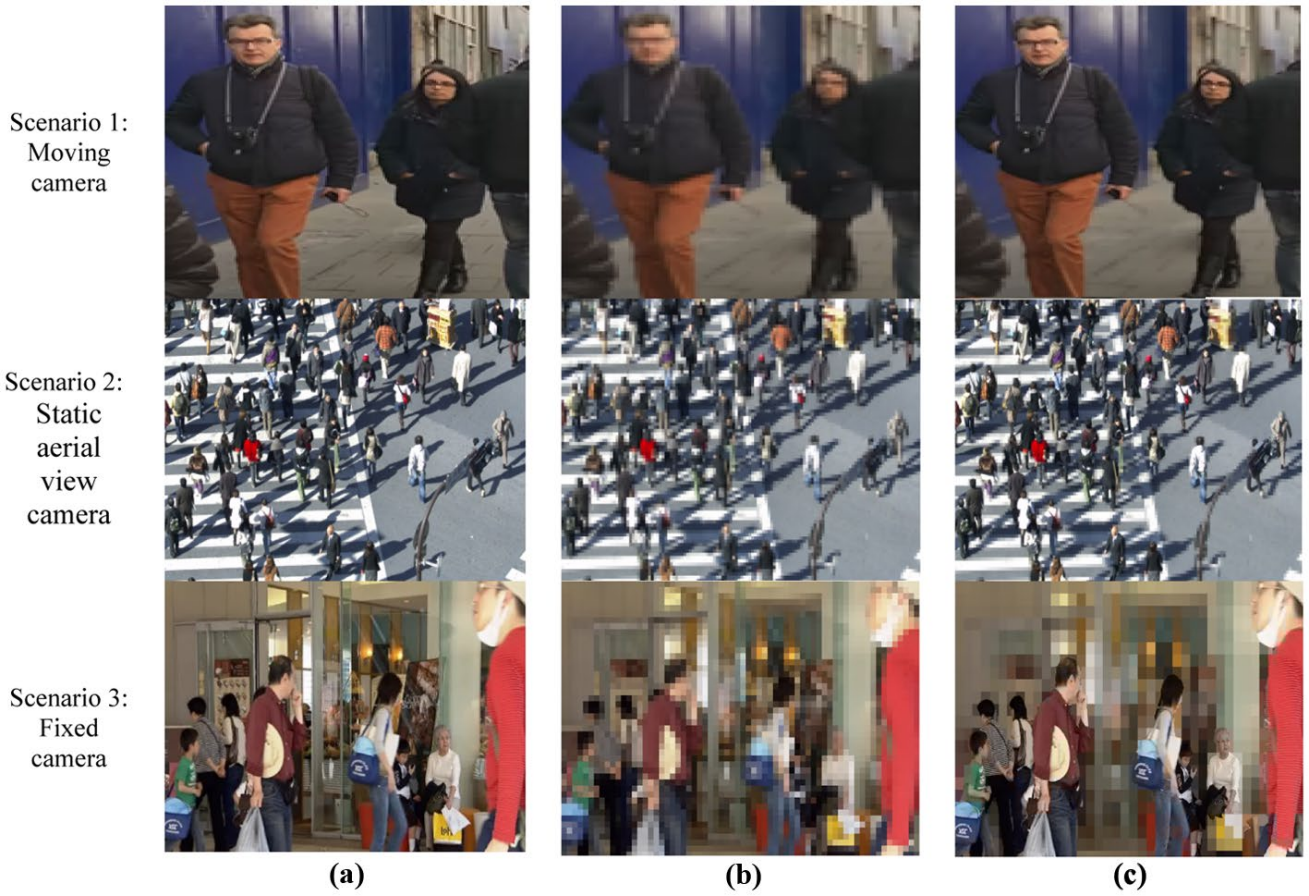
Case study results for smart city surveillance pedestrian scenarios. **a** Original frames **b** Default HEVC **c** ESSE framework

In first case study we conducted the experiments for pedestrian in smart city environment. For testing our proposed framework, we have conducted experiments for three different scenarios for pedestrian. We have utilized testing sequence [12] for scenario 1 where pedestrian and camera both are in dynamic moving positions. Figure 7 shows the results for all three scenarios. In Fig. 7a, scenario 1 presents original extracted frame. Figure 7b scenario 1 presents the encoding results of default HEVC whereas Fig. 7c scenario 1 shows results for proposed ESSE framework. For scenario 1 the achieved PSNR value of default HEVC is 26.50 db and for proposed ESSE is 28.60 db.

In case study 1 scenario 2, we have tested the proposed ESSE for aerial view static camera [23]. Figure 7a scenario 2 represents original extracted frame of crowd from aerial view where the pedestrians are crossing road. Figure 7b scenario 2 shows the encoding result for default HEVC with achieved PSNR of 28.85 db. Figure 7c scenario 2 represents the encoding results for ESSE framework with PSNR value of 30.56 db. In the third scenario we have utilized fixed camera view of indoor building [23]. Figure 7a, scenario 3 shows original extracted frame where varied positioned pedestrian are walking inside a shopping mall. Figure 7b scenario 3 shows the visual results of default HEVC encoding with PSNR value of 27.20 db. Figure 7c shows the results of our proposed ESSE framework for scenario 3 with PSNR value of 29.71 db. The default HEVC results for all three scenario depicts that all the pixels of entire frame are compressed regardless of saliency. Due to which the visual analysis of compressed frame is not possible as the salient features of all pedestrian are lost. On the contrary, from the result of ESSE it can be observed that the surveillance frame encoding is dynamic and the saliency features of pedestrian are preserved in high quality which can be further utilized for visual analysis.

### Case study 2: detection of vehicles

**Fig. 8:**
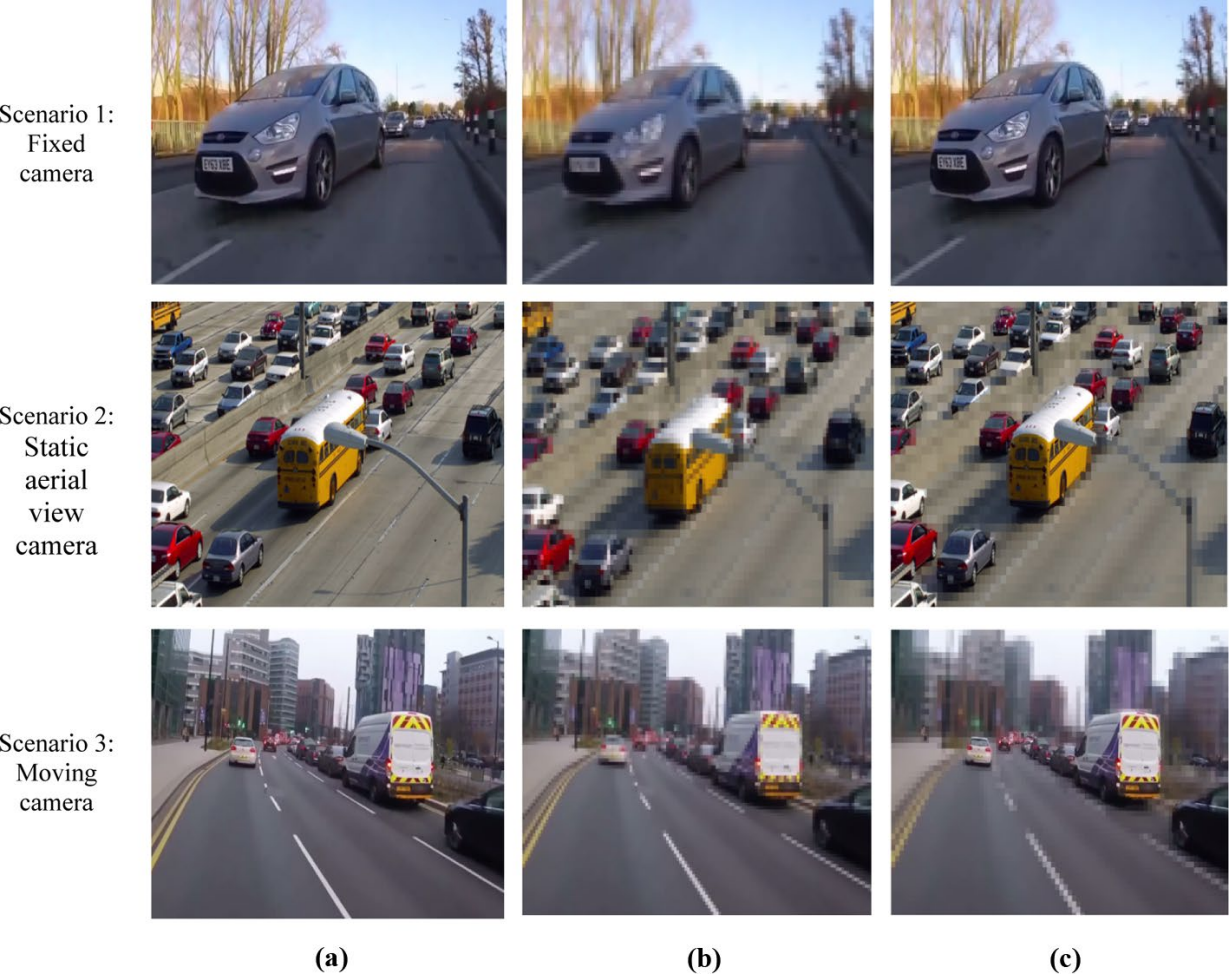
Case study results for smart city surveillance traffic scenarios. **a** Original frames **b** default HEVC **c** ESSE framework

In second case study the experiments are performed for multiple scenario of vehicles on roads in smart city environment. For testing purpose we have utilized testing sequence [9] for static front view camera as fist scenario where a camera is fixed on road side and captures passing by cars. Figure 8a scenario 1 illustrates original extracted frame. The encoding results of default HEVC is depicted from Fig. 8b scenario 1. Figure 8c scenario 1 shows the encoding results of proposed ESSE framework. The achieved PSNR values of default HEVC and proposed ESSE are 29.50 db and 30.49 db respectively.

The second scenario of case study 2 is aerial view static camera [23]. The original extracted frame of scenario 2 is presented in Fig. 8a scenario 2. Figure 8b scenario 2 represents encoding result of default HEVC and Fig. 8c scenario 2 shows the ESSE framework encoding results. The achieved PSNR value of default HEVC is 32.20 db whereas the proposed ESSE achieved higher PSNR value of 31.16 db.

The third scenario of case 2 is moving dash board camera [12]. The results are illustrated in Fig. 8a, scenario 3 which shows original extracted frame where all the vehicles i.e., car, bus, truck are moving on road. The default HEVC and proposed ESSE encoding results are presented in Fig. 8b and c scenario 3. The achieved PSNR value of default HEVC is 29.80 db while the proposed ESSE achieved higher PSNR value of 32.02 db. The overall results depict that the default HEVC compresses entire frame irrespective of salient features of vehicle such as number plates. The visual analysis of default HEVC compressed frame is challenging as the salient features of vehicles are lost during compression. Furthermore, the proposed ESSE framework result shows that it is able to efficiently extract and encode video frames while maintaining the visual quality of salient features of vehicles.

### Case study 3: identification of bicycle/motorbike

**Fig. 9:**
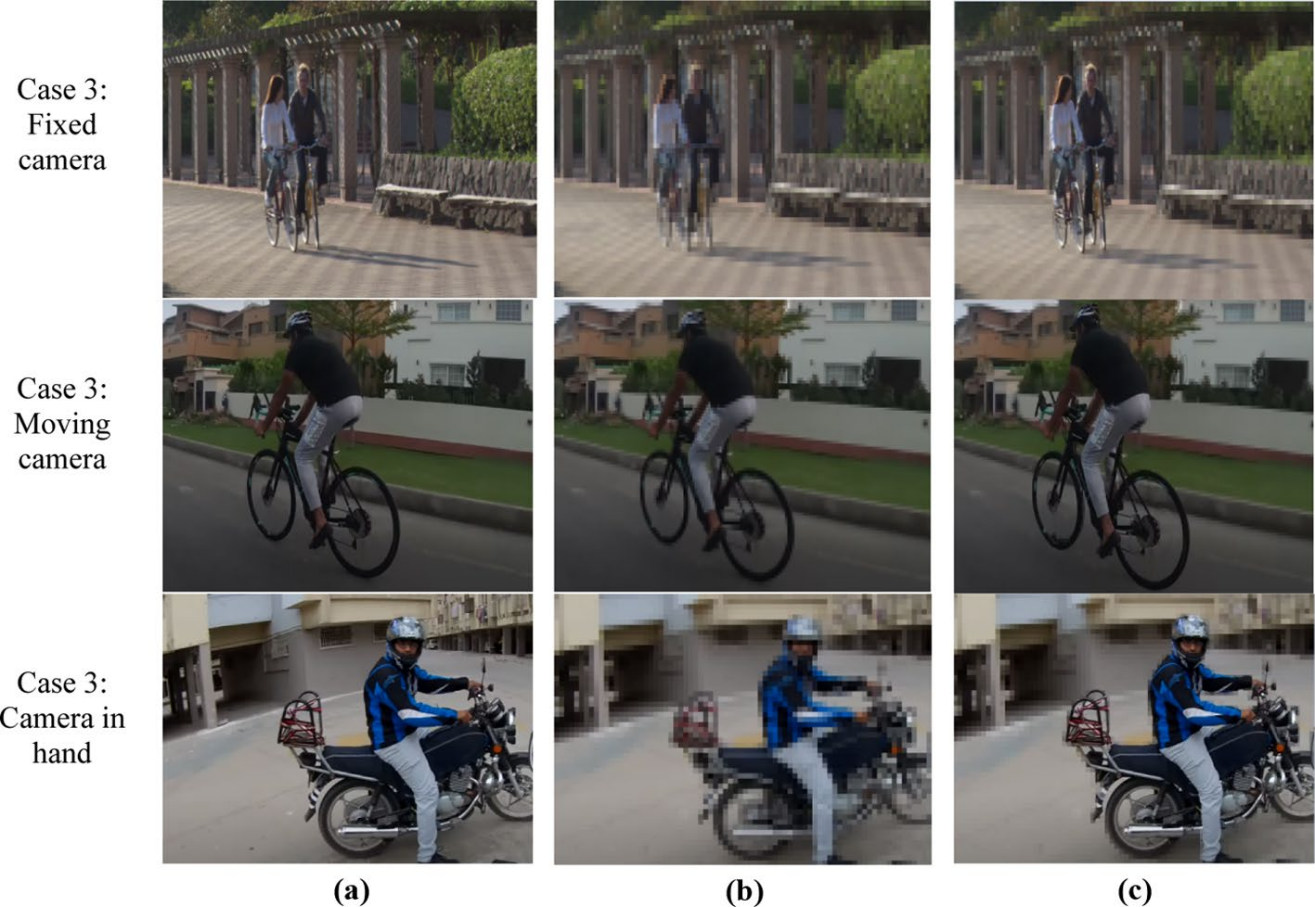
Case study results for smart city surveillance bicycle/motorbike scenarios. **a** Original frames. **b** Default HEVC **c** ESSE framework

The third case study is conducted for different motorbikes and bicycle in smart city environment. The video sequences of static camera [23] is taken for case 3 scenario 1 where camera is static and the multiple bicyclists are in dynamic moving positions. Figure 9 shows the results for all third case study scenarios. In Fig. 9a scenario 1 presents original extracted frame of bicyclists. Figure 9b scenario 1 presents the encoding results of default HEVC whereas Fig. 9c scenario 1 shows results for proposed ESSE framework. For scenario 1 the achieved PSNR value of default HEVC is 31.40 db and for proposed ESSE is 33.4 db.

The second scenario of third case study is a moving camera [12] where the bicyclist and the camera both are in moving position road. Figure 9a scenario 2 illustrates original extracted frame. The encoding results of default HEVC is depicted from Fig. 9b scenario 2. Figure 9c scenario 2 shows the encoding results of proposed ESSE framework. The achieved PSNR values of default HEVC and proposed ESSE are 29.24 db and 30.94 db respectively. The third scenario video sequence is a moving camera in hand [49]. Figure 9a scenario 3, shows extracted frame in which motorcyclist is moving on road.

Figure 9b, c illustrates encoding results of default HEVC and proposed ESSE with achieved PSNR of 28.71 and 30.05 db respectively. As previously discussed results of case 1 and case 2 the results of default HEVC are also similar for case 3 i.e., all the pixels of complete frame are compressed regardless of salient features. Due to which the visual analysis of compressed frame is not possible as the salient features are lost. On the other hand, the results of proposed ESSE framework where the encoding of surveillance video frame is dynamic and the salient features of motorbike and bicycles are preserved in high quality which can be further utilized for visual analysis.

### Comparative discussion

We have evaluated the proposed ESSE framework in term of Pixel accuracy (training and validation), number of parameters, mIoU, bit-rate and PSNR. We have compared the proposed S-SSN with deep learning based state of the art techniques such as Segnet, U-Net and DN which are trained on two different benchmark datasets CamVid and Mapillary vistas for five different classes i.e., pedestrian, vehicle, road, cyclist and motorcyclist. The result elucidates from Figs. 4 and 5 that the proposed S-SSN achieved significant results in terms of mIoU, Pixel accuracy (training and validation) and number of parameters compared with all other techniques.

The overall performance of proposed ESSE framework is also compared with state of the art techniques i.e., perceptual based HEVC and default HEVC. The results are explained in Figs. 2, 3 and Tables 3, 4 depicts that the proposed ESSE performed better and achieved higher PSNR in contrast with low bit-rate. Furthermore, to examine the efficacy of proposed ESSE we have conducted real-time experiments based on case study scenarios. We have considered three environment conditions (1) Ariel view of people on roads, moving camera with people indoor and on footpath with fixed camera. (2) Vehicles captured with fixed camera, Ariel view of vehicles and vehicles captures with moving camera. (3) Cyclists captured by fixed camera, cyclist captured by moving camera and motorcyclist with camera in hand. The case study results demonstrate that the proposed ESSE framework is affective and applicable in numerous environment conditions.

## Conclusions and future work

In a smart city surveillance scenario, the visual data collected through camera is required to be transmitted in high visual quality for analysis. The transmission of high quality visual data is a challenging task on low capacity bandwidth communication channels. To address this issue, we have proposed a surveillance framework ESSE, that integrates deep learning based salient-region (S-R) extraction and efficient video encoding. The ESSE is presented in two phases. In Phase-I, a shallow-semantic segmentation network (S-SSN) is proposed to extract S-Rs and validated for two benchmark datasets. The phase-II generates S-R based salient map, which helps to create a QP map for saliency-based HEVC encoding. The proposed S-SSN achieved pixel-level validation accuracy of 96% and 92% for CamVid and Mapillary vistas datasets respectively. The efficacy of proposed ESSE is also evaluated by conducting extensive smart city surveillance case study for diverse road conditions. The detail results of case study analysis are presented in subsection (4.1–4.4) of result and analysis. The major finding of ESSE framework is the capability of extracting S-Rs and transmitting them in high quality while maintain low bit-rate. Therefore, the proposed solution has a wide applicability for areas having low bandwidth resources. The limitation of this proposed solution is that the training phase of ESSE framework is based on supervised learning. In future, we shall consider the DL semantic segmentation method based on un-supervised learning for Multi-View HEVC [33] to detect the crowded areas to timely ensure social distancing at large scale to avoid massive spread of Covid-19. Moreover, our scheme is efficient for the road scenario but the sensitive data sharing demands to guard against security issues where block-chain may be considered in future to provide dependable solution.

## Data Availability

The publicly available Mapillary Vistas dataset used in this study are available at: https://www.mapillary.com/dataset/vistas?pKey=2Ix3yvNJY9fwQdZWum3t9g&lat=20&lng=0&z=1.5
